# *catena*-Poly[[(acetato-κ^2^ *O*,*O*′)[2-(4-oxo-1,4-dihydro­quinolin-1-yl)acetato-κ*O* ^1^]copper(II)]-μ-4,4′-bipyridine-κ^2^ *N*:*N*′]

**DOI:** 10.1107/S160053681203303X

**Published:** 2012-07-28

**Authors:** Jun Wang, Chuntao Dai, Jianhua Nie

**Affiliations:** a Zhongshan Polytechnic, Zhongshan, Guangdong 528404, People’s Republic of China

## Abstract

In the title compound, [Cu(C_11_H_8_NO_3_)(CH_3_COO)(C_10_H_8_N_2_)]_*n*_, the Cu^II^ ion is six-coordinated by two N atoms from two 4,4′-bipyridine ligands, four O atoms from one acetate ligand, one 2-(4-oxo-1,4-dihydro­quinolin-1-yl)acetate ligand and one water mol­ecule in a distorted octa­hedral geometry. The 4,4′-bipyridine ligands inter­connect [Cu(C_11_H_8_NO_3_)(CH_3_COO)] units, giving rise to a chain along [010]. These chains are further linked to each other by O—H⋯O hydrogen bonds, leading to a two-dimensional supra­molecular network parallel to (100).

## Related literature

For the structures of similar Cd^II^ and Ag^I^ complexes, see: Wang *et al.* (2010 ▶).
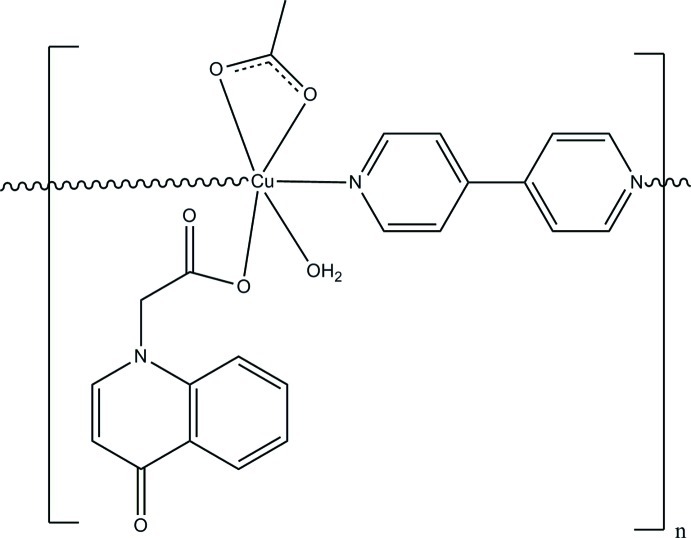


## Experimental

### 

#### Crystal data

[Cu(C_11_H_8_NO_3_)(C_2_H_3_O_2_)(C_10_H_8_N_2_)]
*M* *_r_* = 498.97
Triclinic, 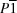

*a* = 9.543 (2) Å
*b* = 11.121 (2) Å
*c* = 11.381 (2) Å
α = 70.03 (3)°
β = 71.27 (3)°
γ = 88.03 (3)°
*V* = 1071.3 (5) Å^3^
*Z* = 2
Mo *K*α radiation
μ = 1.07 mm^−1^
*T* = 298 K
0.35 × 0.26 × 0.22 mm

#### Data collection

Bruker APEXII area-detector diffractometer
Absorption correction: multi-scan (*SADABS*; Sheldrick, 1996 ▶) *T*_min_ = 0.707, *T*_max_ = 0.799
5588 measured reflections
3815 independent reflections
3217 reflections with *I* > 2σ(*I*)
*R*_int_ = 0.018

#### Refinement

*R*[*F* ^2^ > 2σ(*F* ^2^)] = 0.035
*wR*(*F* ^2^) = 0.087
*S* = 1.03
3815 reflections
305 parameters
3 restraints
H atoms treated by a mixture of independent and constrained refinement
Δρ_max_ = 0.27 e Å^−3^
Δρ_min_ = −0.29 e Å^−3^

### 

Data collection: *APEX2* (Bruker, 2004 ▶); cell refinement: *SAINT* (Bruker, 2004 ▶); data reduction: *SAINT*; program(s) used to solve structure: *SHELXS97* (Sheldrick, 2008 ▶); program(s) used to refine structure: *SHELXL97* (Sheldrick, 2008 ▶); molecular graphics: *SHELXTL* (Sheldrick, 2008 ▶); software used to prepare material for publication: *SHELXTL*.

## Supplementary Material

Crystal structure: contains datablock(s) I, global. DOI: 10.1107/S160053681203303X/bg2474sup1.cif

Structure factors: contains datablock(s) I. DOI: 10.1107/S160053681203303X/bg2474Isup2.hkl

Supplementary material file. DOI: 10.1107/S160053681203303X/bg2474Isup3.mol

Additional supplementary materials:  crystallographic information; 3D view; checkCIF report

## Figures and Tables

**Table 1:** Hydrogen-bond geometry (Å, °)

*D*—H⋯*A*	*D*—H	H⋯*A*	*D*⋯*A*	*D*—H⋯*A*
O1*W*—H1*W*⋯O3^i^	0.83 (1)	1.98 (1)	2.795 (3)	166 (3)
O1*W*—H2*W*⋯O2	0.83 (1)	1.99 (2)	2.740 (4)	151 (2)

Symmetry code: (i) 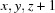.

**Fig. 1.**
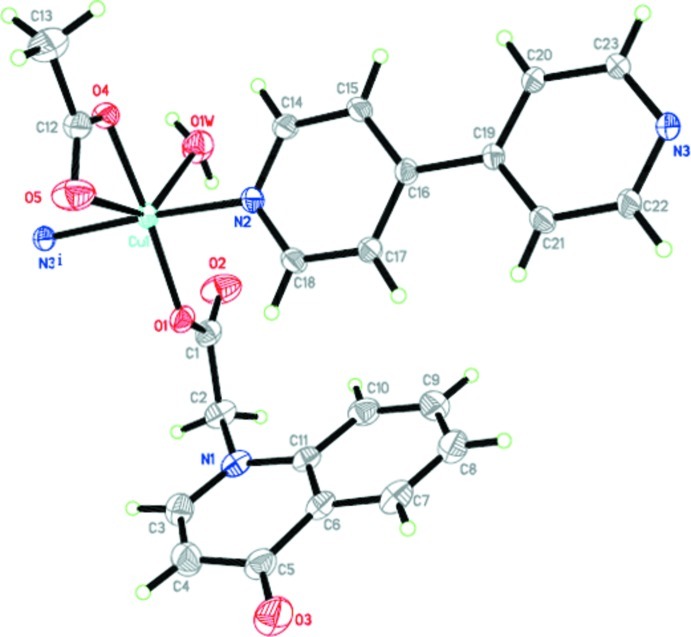
Molecular view of the title compound with displacement ellipsoids drawn at the 30% probability level. Symmetry code: (i) x, 1 + y, z.".

**Fig. 2.**
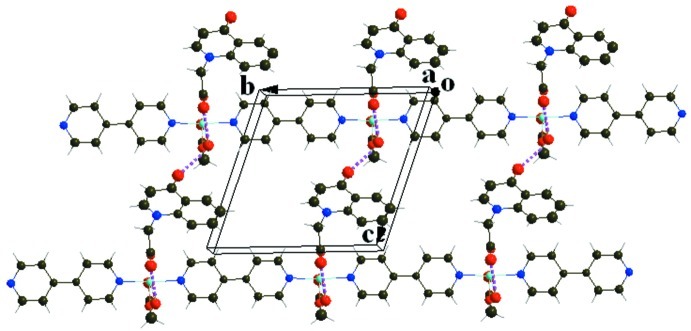
The packing of the title structure.

## supplementary crystallographic information

### Comment

The title compound was obtained upon the reaction of 4-Oxo-1(4*H*) quinolineacetic acid, 4,4'-bipyridine and copper acetate. In the asymmetric unit of the title compound (I) (Fig. 1), each Cu^II^ ion is six-coordinated by two N atoms from two 4,4'-bipyridine ligands, four O atoms from one acetate ligand, one 4-Oxo-1(4*H*)quinolineacetate ligand and one water molecule in a distorted octahedral geometry. The Cu1-O5 bond distance is 2.720 (2)Å, indicative of a weak bond. Similar arrangements are observed in the structures of related mixed-ligand Cd(II) and Ag(I) complexes (Wang *et al.*, 2010). 4,4'-bipyridine ligands interconnect [Cu(CH_3_COO)(C_11_H_8_NO_3_)] moieties, giving rise to a one-dimensional chain along [010]. These chains further link to each other by O—H···O hydrogen bonds (Table 1), leading to a 2D supramolecular network parallel to (100) (Fig. 2).

### Experimental

The title complex was prepared by the addition of a stoichiometric amount of copper acetate (0.181 g; 1 mmol) and 4,4'-bipyridine (0.156 h; 1 mmol) to a hot water/ethanol (*v*/*v* = 1:1) solution (5 ml) of 4-Oxo-1(4*H*) quinolineacetic acid (0.203 g; 1 mmol). The pH was then adjusted to 7.0 to 8.0 with NaOH (10 m*M*/*L*). The resulting solution was filtered, and colorless crystals were obtained at room temperature on slow evaporation of the solvent over several days.

### Refinement

Hydrogen atoms were located in a difference Fourier map. C—H's were further placed at calculated positions (C—H = 0.95–0.99 Å); O—H were refined with restrained O—H = 0.83 (1) Å). In all cases, *U*_iso_(H) were set to 1.2–1.5 times *U*_eq_(C, O).

### Crystal data

**Table:**

[Cu(C_11_H_8_NO_3_)(C_2_H_3_O_2_)(C_10_H_8_N_2_)]	*Z* = 2
*M**_r_* = 498.97	*F*(000) = 514
Triclinic, *P*1	*D*_x_ = 1.547 Mg m^−^^3^
Hall symbol: -P 1	Mo *K*α radiation, λ = 0.71073 Å
*a* = 9.543 (2) Å	Cell parameters from 3600 reflections
*b* = 11.121 (2) Å	θ = 1.3–28.0°
*c* = 11.381 (2) Å	µ = 1.07 mm^−^^1^
α = 70.03 (3)°	*T* = 298 K
β = 71.27 (3)°	Block, blue
γ = 88.03 (3)°	0.35 × 0.26 × 0.22 mm
*V* = 1071.3 (5) Å^3^	

### Data collection

**Table:**

Bruker APEXII area-detector diffractometer	3815 independent reflections
Radiation source: fine-focus sealed tube	3217 reflections with *I* > 2σ(*I*)
Graphite monochromator	*R*_int_ = 0.018
φ and ω scan	θ_max_ = 25.2°, θ_min_ = 2.0°
Absorption correction: multi-scan (*SADABS*; Sheldrick, 1996)	*h* = −6→11
*T*_min_ = 0.707, *T*_max_ = 0.799	*k* = −13→12
5588 measured reflections	*l* = −13→13

### Refinement

**Table:**

Refinement on *F*^2^	Primary atom site location: structure-invariant direct methods
Least-squares matrix: full	Secondary atom site location: difference Fourier map
*R*[*F*^2^ > 2σ(*F*^2^)] = 0.035	Hydrogen site location: inferred from neighbouring sites
*wR*(*F*^2^) = 0.087	H atoms treated by a mixture of independent and constrained refinement
*S* = 1.03	*w* = 1/[σ^2^(*F*_o_^2^) + (0.0368*P*)^2^ + 0.615*P*] where *P* = (*F*_o_^2^ + 2*F*_c_^2^)/3
3815 reflections	(Δ/σ)_max_ < 0.001
305 parameters	Δρ_max_ = 0.27 e Å^−^^3^
3 restraints	Δρ_min_ = −0.29 e Å^−^^3^

### Special details

**Table:**

Geometry. All e.s.d.'s (except the e.s.d. in the dihedral angle between two l.s. planes) are estimated using the full covariance matrix. The cell e.s.d.'s are taken into account individually in the estimation of e.s.d.'s in distances, angles and torsion angles; correlations between e.s.d.'s in cell parameters are only used when they are defined by crystal symmetry. An approximate (isotropic) treatment of cell e.s.d.'s is used for estimating e.s.d.'s involving l.s. planes.
Refinement. Refinement of *F*^2^ against ALL reflections. The weighted *R*-factor *wR* and goodness of fit *S* are based on *F*^2^, conventional *R*-factors *R* are based on *F*, with *F* set to zero for negative *F*^2^. The threshold expression of *F*^2^ > σ(*F*^2^) is used only for calculating *R*-factors(gt) *etc*. and is not relevant to the choice of reflections for refinement. *R*-factors based on *F*^2^ are statistically about twice as large as those based on *F*, and *R*- factors based on ALL data will be even larger.

### Fractional atomic coordinates and isotropic or equivalent isotropic displacement parameters (Å^2^)

**Table:**

	*x*	*y*	*z*	*U*_iso_*/*U*_eq_	
Cu1	0.11865 (4)	0.27261 (3)	0.20437 (3)	0.02973 (11)	
O1	0.2694 (2)	0.32890 (16)	0.02845 (18)	0.0357 (4)	
O2	0.4718 (3)	0.3200 (3)	0.0881 (2)	0.0671 (7)	
O3	0.2324 (3)	0.3238 (3)	−0.4737 (2)	0.0694 (7)	
O4	−0.0535 (2)	0.22122 (16)	0.36609 (17)	0.0357 (4)	
O5	−0.1717 (3)	0.2636 (2)	0.2207 (2)	0.0624 (6)	
N1	0.4365 (3)	0.3819 (2)	−0.2305 (2)	0.0394 (6)	
N2	0.1132 (2)	0.09355 (19)	0.1985 (2)	0.0319 (5)	
N3	0.1123 (2)	−0.54349 (19)	0.1965 (2)	0.0295 (5)	
C1	0.4073 (3)	0.3444 (3)	0.0058 (3)	0.0391 (7)	
C2	0.5017 (3)	0.4057 (3)	−0.1394 (3)	0.0452 (7)	
H2A	0.5156	0.4976	−0.1608	0.054*	
H2B	0.5987	0.3726	−0.1526	0.054*	
C3	0.3660 (4)	0.4747 (3)	−0.2956 (3)	0.0499 (8)	
H3	0.3653	0.5537	−0.2844	0.060*	
C4	0.2970 (4)	0.4590 (3)	−0.3756 (3)	0.0562 (9)	
H4	0.2525	0.5277	−0.4192	0.067*	
C5	0.2900 (3)	0.3401 (3)	−0.3956 (3)	0.0474 (7)	
C6	0.3541 (3)	0.2366 (3)	−0.3155 (3)	0.0395 (7)	
C7	0.3422 (3)	0.1112 (3)	−0.3155 (3)	0.0494 (8)	
H7	0.2886	0.0941	−0.3642	0.059*	
C8	0.4068 (4)	0.0137 (3)	−0.2466 (3)	0.0559 (9)	
H8	0.3966	−0.0689	−0.2472	0.067*	
C9	0.4884 (4)	0.0404 (3)	−0.1749 (3)	0.0559 (9)	
H9	0.5364	−0.0246	−0.1304	0.067*	
C10	0.4991 (3)	0.1599 (3)	−0.1692 (3)	0.0474 (7)	
H10	0.5523	0.1751	−0.1193	0.057*	
C11	0.4307 (3)	0.2601 (3)	−0.2377 (3)	0.0377 (6)	
C12	−0.1719 (3)	0.2234 (3)	0.3354 (3)	0.0393 (7)	
C13	−0.3133 (4)	0.1725 (3)	0.4495 (3)	0.0604 (9)	
H13A	−0.3963	0.1882	0.4175	0.091*	
H13B	−0.3225	0.2150	0.5119	0.091*	
H13C	−0.3117	0.0819	0.4919	0.091*	
C14	0.0857 (3)	−0.0086 (2)	0.3104 (3)	0.0402 (7)	
H14	0.0704	0.0056	0.3894	0.048*	
C15	0.0791 (3)	−0.1324 (2)	0.3141 (3)	0.0399 (7)	
H15	0.0585	−0.1998	0.3946	0.048*	
C16	0.1030 (3)	−0.1582 (2)	0.1983 (2)	0.0282 (5)	
C17	0.1312 (3)	−0.0516 (2)	0.0827 (3)	0.0324 (6)	
H17	0.1474	−0.0631	0.0023	0.039*	
C18	0.1352 (3)	0.0702 (2)	0.0862 (3)	0.0345 (6)	
H18	0.1539	0.1395	0.0074	0.041*	
C19	0.1027 (3)	−0.2911 (2)	0.1972 (2)	0.0272 (5)	
C20	0.0947 (3)	−0.3960 (2)	0.3098 (3)	0.0341 (6)	
H20	0.0870	−0.3833	0.3882	0.041*	
C21	0.1165 (3)	−0.3182 (2)	0.0836 (3)	0.0370 (6)	
H21	0.1219	−0.2519	0.0053	0.044*	
C22	0.1220 (3)	−0.4430 (2)	0.0868 (3)	0.0363 (6)	
H22	0.1331	−0.4584	0.0091	0.044*	
C23	0.0982 (3)	−0.5181 (2)	0.3063 (3)	0.0346 (6)	
H23	0.0903	−0.5864	0.3838	0.041*	
O1W	0.2742 (3)	0.2058 (2)	0.3379 (2)	0.0547 (6)	
H1W	0.271 (4)	0.232 (3)	0.399 (2)	0.082*	
H2W	0.355 (2)	0.232 (4)	0.278 (2)	0.082*	

### Atomic displacement parameters (Å^2^)

**Table:**

	*U*^11^	*U*^22^	*U*^33^	*U*^12^	*U*^13^	*U*^23^
Cu1	0.0389 (2)	0.02133 (17)	0.03041 (19)	0.00295 (13)	−0.00988 (14)	−0.01232 (13)
O1	0.0385 (11)	0.0323 (10)	0.0359 (10)	−0.0001 (8)	−0.0065 (9)	−0.0165 (8)
O2	0.0590 (15)	0.103 (2)	0.0461 (14)	0.0028 (14)	−0.0246 (12)	−0.0273 (13)
O3	0.0803 (18)	0.0945 (19)	0.0690 (16)	0.0325 (15)	−0.0506 (15)	−0.0497 (15)
O4	0.0438 (11)	0.0315 (10)	0.0302 (10)	0.0017 (8)	−0.0093 (9)	−0.0115 (8)
O5	0.0623 (15)	0.0840 (17)	0.0379 (13)	0.0036 (13)	−0.0206 (12)	−0.0136 (12)
N1	0.0356 (13)	0.0506 (14)	0.0342 (13)	−0.0017 (11)	−0.0076 (11)	−0.0207 (11)
N2	0.0425 (13)	0.0232 (11)	0.0305 (12)	0.0020 (10)	−0.0107 (10)	−0.0111 (9)
N3	0.0350 (12)	0.0236 (10)	0.0303 (12)	0.0026 (9)	−0.0091 (10)	−0.0114 (9)
C1	0.0439 (17)	0.0395 (15)	0.0404 (16)	0.0009 (13)	−0.0137 (14)	−0.0219 (13)
C2	0.0376 (16)	0.0578 (19)	0.0428 (17)	−0.0086 (14)	−0.0091 (14)	−0.0233 (15)
C3	0.056 (2)	0.0464 (18)	0.052 (2)	0.0063 (16)	−0.0170 (17)	−0.0237 (15)
C4	0.064 (2)	0.057 (2)	0.059 (2)	0.0206 (17)	−0.0325 (19)	−0.0248 (17)
C5	0.0435 (17)	0.066 (2)	0.0431 (17)	0.0159 (15)	−0.0192 (15)	−0.0278 (16)
C6	0.0339 (15)	0.0539 (18)	0.0357 (15)	0.0067 (13)	−0.0089 (13)	−0.0243 (14)
C7	0.0439 (18)	0.065 (2)	0.0473 (19)	0.0033 (16)	−0.0119 (15)	−0.0328 (17)
C8	0.065 (2)	0.0495 (19)	0.052 (2)	0.0059 (17)	−0.0100 (18)	−0.0242 (16)
C9	0.063 (2)	0.053 (2)	0.0460 (19)	0.0169 (17)	−0.0156 (17)	−0.0135 (16)
C10	0.0442 (18)	0.063 (2)	0.0396 (17)	0.0089 (15)	−0.0177 (15)	−0.0200 (15)
C11	0.0330 (15)	0.0481 (16)	0.0300 (14)	0.0026 (13)	−0.0043 (12)	−0.0167 (13)
C12	0.0459 (17)	0.0339 (15)	0.0349 (16)	0.0025 (13)	−0.0085 (13)	−0.0125 (12)
C13	0.051 (2)	0.070 (2)	0.051 (2)	−0.0039 (18)	−0.0039 (17)	−0.0207 (18)
C14	0.064 (2)	0.0297 (14)	0.0273 (14)	0.0032 (13)	−0.0118 (14)	−0.0138 (12)
C15	0.067 (2)	0.0244 (13)	0.0267 (14)	0.0032 (13)	−0.0134 (14)	−0.0086 (11)
C16	0.0316 (14)	0.0248 (13)	0.0306 (14)	0.0030 (11)	−0.0109 (11)	−0.0121 (11)
C17	0.0465 (16)	0.0278 (13)	0.0275 (14)	0.0048 (12)	−0.0149 (12)	−0.0127 (11)
C18	0.0491 (17)	0.0243 (13)	0.0302 (14)	0.0019 (12)	−0.0152 (13)	−0.0074 (11)
C19	0.0307 (14)	0.0231 (12)	0.0290 (14)	0.0035 (10)	−0.0099 (11)	−0.0104 (10)
C20	0.0492 (17)	0.0281 (13)	0.0265 (14)	0.0058 (12)	−0.0106 (12)	−0.0132 (11)
C21	0.0605 (19)	0.0248 (13)	0.0293 (14)	0.0065 (13)	−0.0207 (14)	−0.0084 (11)
C22	0.0569 (18)	0.0272 (13)	0.0325 (15)	0.0066 (13)	−0.0206 (14)	−0.0146 (11)
C23	0.0470 (16)	0.0246 (13)	0.0270 (14)	0.0046 (12)	−0.0074 (12)	−0.0075 (11)
O1W	0.0623 (15)	0.0609 (14)	0.0543 (14)	0.0106 (12)	−0.0273 (12)	−0.0295 (12)

### Geometric parameters (Å, º)

**Table:**

Cu1—O4	1.954 (2)	C7—H7	0.9300
Cu1—O1	1.956 (2)	C8—C9	1.396 (5)
Cu1—N3^i^	2.016 (2)	C8—H8	0.9300
Cu1—N2	2.018 (2)	C9—C10	1.362 (4)
Cu1—O1W	2.381 (2)	C9—H9	0.9300
Cu1—O5	2.720 (2)	C10—C11	1.399 (4)
O1—C1	1.263 (3)	C10—H10	0.9300
O2—C1	1.231 (3)	C12—C13	1.501 (4)
O3—C5	1.247 (3)	C13—H13A	0.9600
O4—C12	1.282 (3)	C13—H13B	0.9600
O5—C12	1.226 (3)	C13—H13C	0.9600
N1—C3	1.347 (4)	C14—C15	1.366 (3)
N1—C11	1.389 (3)	C14—H14	0.9300
N1—C2	1.464 (3)	C15—C16	1.391 (3)
N2—C18	1.340 (3)	C15—H15	0.9300
N2—C14	1.341 (3)	C16—C17	1.392 (3)
N3—C23	1.338 (3)	C16—C19	1.482 (3)
N3—C22	1.340 (3)	C17—C18	1.371 (3)
N3—Cu1^ii^	2.016 (2)	C17—H17	0.9300
C1—C2	1.524 (4)	C18—H18	0.9300
C2—H2A	0.9700	C19—C20	1.390 (3)
C2—H2B	0.9700	C19—C21	1.391 (3)
C3—C4	1.340 (4)	C20—C23	1.371 (3)
C3—H3	0.9300	C20—H20	0.9300
C4—C5	1.424 (4)	C21—C22	1.376 (3)
C4—H4	0.9300	C21—H21	0.9300
C5—C6	1.457 (4)	C22—H22	0.9300
C6—C7	1.404 (4)	C23—H23	0.9300
C6—C11	1.405 (4)	O1W—H1W	0.830 (10)
C7—C8	1.361 (4)	O1W—H2W	0.827 (10)
			
O4—Cu1—O1	171.44 (8)	C8—C9—H9	119.4
O4—Cu1—N3^i^	90.50 (9)	C9—C10—C11	120.6 (3)
O1—Cu1—N3^i^	89.24 (9)	C9—C10—H10	119.7
O4—Cu1—N2	90.29 (9)	C11—C10—H10	119.7
O1—Cu1—N2	89.09 (9)	N1—C11—C10	121.7 (3)
N3^i^—Cu1—N2	173.97 (8)	N1—C11—C6	119.2 (3)
O4—Cu1—O1W	88.68 (8)	C10—C11—C6	119.1 (3)
O1—Cu1—O1W	99.84 (8)	O5—C12—O4	123.0 (3)
N3^i^—Cu1—O1W	97.50 (8)	O5—C12—C13	121.2 (3)
N2—Cu1—O1W	88.49 (9)	O4—C12—C13	115.8 (3)
C1—O1—Cu1	125.00 (18)	C12—C13—H13A	109.5
C12—O4—Cu1	109.13 (16)	C12—C13—H13B	109.5
C3—N1—C11	119.7 (2)	H13A—C13—H13B	109.5
C3—N1—C2	119.5 (2)	C12—C13—H13C	109.5
C11—N1—C2	120.4 (2)	H13A—C13—H13C	109.5
C18—N2—C14	117.0 (2)	H13B—C13—H13C	109.5
C18—N2—Cu1	122.92 (17)	N2—C14—C15	123.3 (2)
C14—N2—Cu1	120.03 (17)	N2—C14—H14	118.4
C23—N3—C22	117.0 (2)	C15—C14—H14	118.4
C23—N3—Cu1^ii^	119.02 (17)	C14—C15—C16	120.4 (2)
C22—N3—Cu1^ii^	124.00 (17)	C14—C15—H15	119.8
O2—C1—O1	127.0 (3)	C16—C15—H15	119.8
O2—C1—C2	117.2 (3)	C15—C16—C17	115.9 (2)
O1—C1—C2	115.7 (2)	C15—C16—C19	122.1 (2)
N1—C2—C1	113.5 (2)	C17—C16—C19	121.9 (2)
N1—C2—H2A	108.9	C18—C17—C16	120.7 (2)
C1—C2—H2A	108.9	C18—C17—H17	119.7
N1—C2—H2B	108.9	C16—C17—H17	119.7
C1—C2—H2B	108.9	N2—C18—C17	122.7 (2)
H2A—C2—H2B	107.7	N2—C18—H18	118.6
C4—C3—N1	123.5 (3)	C17—C18—H18	118.6
C4—C3—H3	118.3	C20—C19—C21	116.1 (2)
N1—C3—H3	118.3	C20—C19—C16	121.3 (2)
C3—C4—C5	121.8 (3)	C21—C19—C16	122.6 (2)
C3—C4—H4	119.1	C23—C20—C19	120.5 (2)
C5—C4—H4	119.1	C23—C20—H20	119.7
O3—C5—C4	123.5 (3)	C19—C20—H20	119.7
O3—C5—C6	122.0 (3)	C22—C21—C19	120.1 (2)
C4—C5—C6	114.5 (3)	C22—C21—H21	119.9
C7—C6—C11	118.4 (3)	C19—C21—H21	119.9
C7—C6—C5	120.7 (3)	N3—C22—C21	123.2 (2)
C11—C6—C5	120.9 (3)	N3—C22—H22	118.4
C8—C7—C6	122.1 (3)	C21—C22—H22	118.4
C8—C7—H7	119.0	N3—C23—C20	123.1 (2)
C6—C7—H7	119.0	N3—C23—H23	118.5
C7—C8—C9	118.6 (3)	C20—C23—H23	118.5
C7—C8—H8	120.7	Cu1—O1W—H1W	124 (3)
C9—C8—H8	120.7	Cu1—O1W—H2W	97 (3)
C10—C9—C8	121.2 (3)	H1W—O1W—H2W	109.3 (17)
C10—C9—H9	119.4		
			
N3^i^—Cu1—O1—C1	85.9 (2)	C3—N1—C11—C6	1.5 (4)
N2—Cu1—O1—C1	−99.9 (2)	C2—N1—C11—C6	173.5 (3)
O1W—Cu1—O1—C1	−11.6 (2)	C9—C10—C11—N1	178.2 (3)
N3^i^—Cu1—O4—C12	92.99 (17)	C9—C10—C11—C6	−1.7 (4)
N2—Cu1—O4—C12	−81.03 (17)	C7—C6—C11—N1	−176.4 (2)
O1W—Cu1—O4—C12	−169.51 (17)	C5—C6—C11—N1	4.3 (4)
O4—Cu1—N2—C18	138.5 (2)	C7—C6—C11—C10	3.5 (4)
O1—Cu1—N2—C18	−33.0 (2)	C5—C6—C11—C10	−175.8 (3)
O1W—Cu1—N2—C18	−132.8 (2)	Cu1—O4—C12—O5	−6.1 (3)
O4—Cu1—N2—C14	−41.1 (2)	Cu1—O4—C12—C13	173.6 (2)
O1—Cu1—N2—C14	147.5 (2)	C18—N2—C14—C15	−0.1 (4)
O1W—Cu1—N2—C14	47.6 (2)	Cu1—N2—C14—C15	179.4 (2)
Cu1—O1—C1—O2	4.8 (4)	N2—C14—C15—C16	0.7 (5)
Cu1—O1—C1—C2	−172.18 (17)	C14—C15—C16—C17	−0.8 (4)
C3—N1—C2—C1	101.4 (3)	C14—C15—C16—C19	177.5 (3)
C11—N1—C2—C1	−70.6 (3)	C15—C16—C17—C18	0.3 (4)
O2—C1—C2—N1	155.2 (3)	C19—C16—C17—C18	−178.0 (2)
O1—C1—C2—N1	−27.5 (4)	C14—N2—C18—C17	−0.4 (4)
C11—N1—C3—C4	−4.6 (5)	Cu1—N2—C18—C17	−179.9 (2)
C2—N1—C3—C4	−176.6 (3)	C16—C17—C18—N2	0.2 (4)
N1—C3—C4—C5	1.5 (6)	C15—C16—C19—C20	−7.2 (4)
C3—C4—C5—O3	−176.7 (3)	C17—C16—C19—C20	171.1 (3)
C3—C4—C5—C6	4.1 (5)	C15—C16—C19—C21	175.5 (3)
O3—C5—C6—C7	−5.3 (5)	C17—C16—C19—C21	−6.2 (4)
C4—C5—C6—C7	173.8 (3)	C21—C19—C20—C23	−1.0 (4)
O3—C5—C6—C11	174.0 (3)	C16—C19—C20—C23	−178.4 (2)
C4—C5—C6—C11	−6.9 (4)	C20—C19—C21—C22	−0.3 (4)
C11—C6—C7—C8	−2.4 (4)	C16—C19—C21—C22	177.2 (3)
C5—C6—C7—C8	176.9 (3)	C23—N3—C22—C21	−0.7 (4)
C6—C7—C8—C9	−0.7 (5)	Cu1^ii^—N3—C22—C21	179.5 (2)
C7—C8—C9—C10	2.6 (5)	C19—C21—C22—N3	1.1 (4)
C8—C9—C10—C11	−1.4 (5)	C22—N3—C23—C20	−0.6 (4)
C3—N1—C11—C10	−178.4 (3)	Cu1^ii^—N3—C23—C20	179.2 (2)
C2—N1—C11—C10	−6.5 (4)	C19—C20—C23—N3	1.5 (4)

Symmetry codes: (i) *x*, *y*+1, *z*; (ii) *x*, *y*−1, *z*.

### Hydrogen-bond geometry (Å, º)

**Table:**

*D*—H···*A*	*D*—H	H···*A*	*D*···*A*	*D*—H···*A*
O1*W*—H1*W*···O3^iii^	0.83 (1)	1.98 (1)	2.795 (3)	166 (3)
O1*W*—H2*W*···O2	0.83 (1)	1.99 (2)	2.740 (4)	151 (2)

Symmetry code: (iii) *x*, *y*, *z*+1.
